# Repeatability of Quantitative Imaging Features in Prostate Magnetic Resonance Imaging

**DOI:** 10.3389/fonc.2020.00551

**Published:** 2020-05-07

**Authors:** Hong Lu, Nestor A. Parra, Jin Qi, Kenneth Gage, Qian Li, Shuxuan Fan, Sebastian Feuerlein, Julio Pow-Sang, Robert Gillies, Jung W. Choi, Yoganand Balagurunathan

**Affiliations:** ^1^Department of Radiology, Tianjin Medical and Cancer Hospital, Tianjin, China; ^2^Departments of Cancer Physiology, H. Lee Moffitt Cancer Center, Tampa, FL, United States; ^3^Departments of Diagnostic Imaging, H. Lee Moffitt Cancer Center, Tampa, FL, United States; ^4^Departments of Genitourinary Oncology, H. Lee Moffitt Cancer Center, Tampa, FL, United States; ^5^Departments of Bioinformatics & Biostatistics, H. Lee Moffitt Cancer Center, Tampa, FL, United States

**Keywords:** radiomics, mpMRI, prostate cancer, test–retest in mpMRI, prostate TRUS-MRI, repeatable MRI features

## Abstract

**Background:** Multiparametric magnetic resonance imaging (mpMRI) has emerged as a non-invasive modality to diagnose and monitor prostate cancer. Quantitative metrics on the regions of abnormality have shown to be useful descriptors to discriminate clinically significant cancers. In this study, we evaluate the reproducibility of quantitative imaging features using repeated mpMRI on the same patients.

**Methods:** We retrospectively obtained the deidentified records of patients, who underwent two mpMRI scans within 2 weeks of the first baseline scan. The patient records were obtained as deidentified data (including imaging), obtained through the TCIA (The Cancer Imaging Archive) repository and analyzed in our institution with an institutional review board–approved Health Insurance Portability and Accountability Act–compliant retrospective study protocol. Indicated biopsied regions were used as a marker for our study radiologists to delineate the regions of interest. We extracted 307 quantitative features in each mpMRI modality [T2-weighted MR sequence image (T2w) and apparent diffusion coefficient (ADC) with *b* values of 0 and 1,400 mm/s^2^] across the two sequential scans. Concordance correlation coefficients (CCCs) were computed on the features extracted from sequential scans. Redundant features were removed by computing the coefficient of determination (*R*^2^) among them and replaced with a feature that had the highest dynamic range within intercorrelated groups.

**Results:** We have assessed the reproducibility of quantitative imaging features among sequential scans and found that habitat region characterization improves repeatability in ADC maps. There were 19 T2w features and two ADC features in radiologist drawn regions (native raw image), compared to 18 T2w and 15 ADC features in habitat regions (sphere), which were reproducible (CCC ≥0.65) and non-redundant (*R*^2^ ≥ 0.99). We also found that *z*-transformation of the images prior to feature extraction reduced the number of reproducible features with no detrimental effect.

**Conclusion:** We have shown that there are quantitative imaging features that are reproducible across sequential prostate mpMRI acquisition at a preset level of filters. We also found that a habitat approach improves feature repeatability in ADC. A validated set of reproducible image features in mpMRI will allow us to develop clinically useful disease risk stratification, enabling the possibility of using imaging as a surrogate to invasive biopsies.

## Introduction

Prostate cancer detection using multiparametric magnetic resonance imaging (mpMRI) has been gaining consensus in the community for disease detection due to superior lesion sensitivity compared to transrectal ultrasound (TRUS) imaging (1, 2). Multiparametric MRI modalities have been useful in estimating size, volume, and relation to the underlying pathology of prostate cancer (3). Improvements in imaging technologies coupled with advances in mpMRI have led to its combined use with TRUS to guide prostate biopsies that improve detection of clinically aggressive cancers (4). Most clinical diagnoses follow a consensus reporting standard with the adoption of Prostate Imaging Reporting and Data Systems (PI-RADS v2) (5), which provides qualitative guidelines for clinical assessment. Variability in mpMRI scan interpretations among radiologists can in part be attributed to the steep learning curve required to interpret the scans (6). Quantitative imaging metrics or radiomics has been used to distinguish clinical abnormalities found in medical imaging (7, 8). For example, radiomics has been shown to be both reproducible in lung cancer computed tomography imaging and prognostic of lung cancer patient survival (9, 10). Recently, quantitative imaging features obtained from tumor regions on prostate mpMRI scans have been shown to be both predictive of clinically aggressive disease (11) and improve PI-RADS performance (12). In a recent survey on the role of imaging biomarkers in clinical decision making, the European Organization for Research and Treatment of Cancer and Cancer Research UK released a consensus statement with key recommendations to accelerate clinical biomarker translation (13). The key component of the consensus statement emphasizes the importance of validating the repeatability and reproducibility of these biomarkers for.

Information extraction (as part of a technical assay) and for proper downstream clinical utilization. Repeatability and reproducibility are necessary, but not sufficient, conditions for clinical usage of imaging biomarkers, as there is a higher relevance requirement such as accurate cancer prediction and prognosis (14, 15). As mpMRI has no biological reference for derived image intensity values, there are studies that have proposed standardizing these values (16–18). Recently, there have been efforts to find repeatable quantitative (radiomic) features in mpMRI scan of various cancers, such as rectal (19), cervix (20), lacrimal gland (21), and prostate (22, 23). Notably in prostate (23) and cervical studies (20), enrolled patients were scanned in a test–retest setting. Quantification of regions of interest has been accomplished in various ways, either through the use of a few open source tools (24) or more commonly through custom implementation methods. Recently, there has been an initiative to standardize definitions of these quantitative metrics, as recommended by the Image Biomarker Standardization Initiative (IBSI) (25). In our study, we obtained test–retest deidentified prostate mpMRI studies from patients enrolled at the Brigham and Women's Hospital, shared in a public repository (26). Patients with pathologically verified lesions were scored by a clinical pathologist (Gleason score). Independently marked regions of interest were standardized and quantified using custom radiomic features that followed the IBSI consensus criteria (25). We investigated the feasibility of reproducing these features across the cohort for a diverse set of prostate lesions. We also propose a habitat-based approach that converges regions of interest, followed by lesion characterization to improve repeatability of image features. This work will provide the basis for using repeatable quantitative features in prognostic evaluation of prostate cancer patients.

## Materials and Methods

We obtained deidentified mpMRI patient images along with segmentation masks (Dicom-Seg) through The Cancer Imaging Archive (TCIA) collection titled “QIN-PROSTATE-Repeatability” with detailed descriptions summarized about the cohort (26).

**Figure 1:**
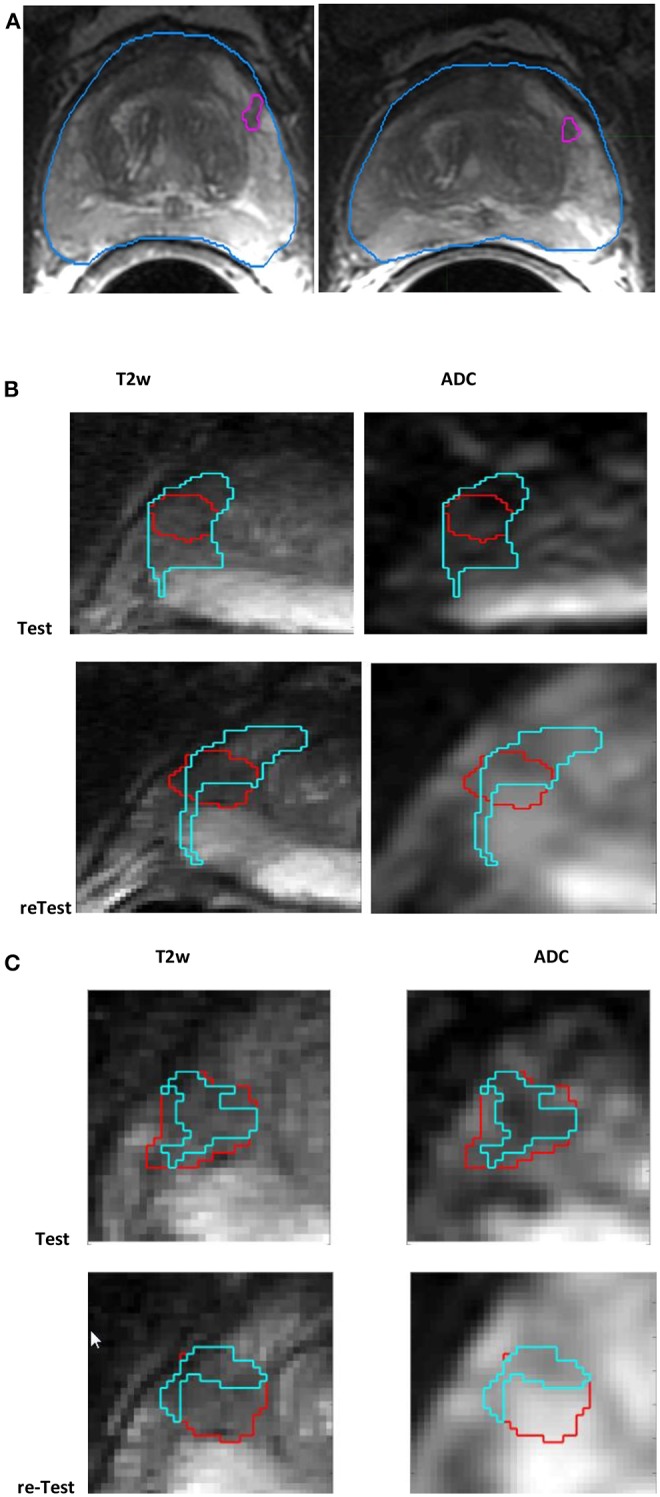
Screen capture of mpMRI prostate scan **(A)** with radiologist-marked lesion shown for the baseline and follow-up scans in T2w, **(B)** habitat converged with a sphere (15mm), and **(C)** habitat (≤ median) (in cyan) for a lesion (in red) shown for test and retest (along the rows) in T2w, ADC (along the column).

The patients were accrued for a research study at Brigham and Women's Hospital, Harvard Medical School. The patients waived informed consent, and their deidentified records were analyzed through our institutional review board–approved Health Insurance Portability and Accountability Act–compliant retrospective study protocol. The original study collection had 15 treatment-naive men who had mpMRI scans and biopsy-confirmed pathology and were scanned again within 2 weeks of their first baseline scan, during which patients did not receive any interim treatment. The cohort had 11 patients with a standard template biopsy and four patients who had suspicion of prostate cancer based on their clinical record. The mpMRI scans had T2w axial images [repetition time (TR) 3,350–5,109 ms, time to echo (TE) 84–107 ms, field of view (FOV) 140–200 nm] and ADC map derived from the diffusion-weighted MRI (*b* = 0, 1,400 s/mm, TR 2,500–8,250 ms, TE 76–80 ms, FOV 760–280mm). Figure 1 illustrates sample lesions delineated on the test and retest T2w axial images.

Our study radiologist (H.L.) read the patient scans and localized lesions within the regions on patient scans identified by the prior study and provided consensus region segmentation in consultation with the second study radiologist (J.Q.). A third radiologist (K.G.) provided a random overread. Our radiologists in consensus agreed to use 13 of the 15 patient mpMRIs; scans from two patients were dropped because of disagreements in identifying the abnormality and suboptimal quality of the scans. Among the converged patients, our radiologists in consensus identified 15 tumor lesions that were anatomically matched, longitudinally, across the test and retest time images. Of these, 11 identified lesion boundaries were verified to match with the prior study at the same anatomical location that had been pathologically verified (Gleason score); in addition, four additional lesion abnormalities were identified by our study radiologist(s), matched longitudinally. Table 1 provides patient clinical details, including subject identifier, lesions anatomical location, prostate-specific antigen (PSA) value, and pathological diagnostic (Gleason) score. Newly identified lesions without corroborating pathological findings were marked as not available (NA), by our study radiologists.

**Table 1:** Summary of patient scans with clinical diagnosis for the biopsies.

**#**	**Subject**	**MRI exam**	**PSA, ng/mL**	**Gleason(Bx)**	**Gland**	**PI-RADS v2 (test)**	**PI_RADS v2 (retest)**	**Comment**
1	1-b1	Known Pca, staging	5.4	3 + 4	PZ	4	4	Identified
2	1-b2			NA	PZ	4	4	Additional
3	2-b1	Known Pca, assess change	7.5	3 + 4	PZ	2	3	Identified
4	2-b2			NA	PZ	2	3	Additional
5	3	Known Pca, staging	8.2	3 + 3	PZ	4	4	Identified
6	4	Known Pca, staging	4.3	3 + 3	PZ/TZ	2	2	Identified
7	5-b2	Suspected Pca, staging	NA	NA	TZ	2	2	Additional
8	6-b2	Elevated PSA, staging	5	Benign	TZ	1	1	Additional
9	7	Elevated PSA, staging	6.2	4 + 5	PZ	4	4	Identified
10	8	Known Pca, assess change	4.8	4.8	PZ	4	4	Identified
11	9	Elevated PSA, staging	9.4	Benign	PZ	4	4	Identified
12	10	Known Pca, assess change	3.15	3 + 3	PZ	4	4	Identified
13	11	Known Pca, assess change	9.7	3 + 3	PZ	4	4	Identified
14	12	Elevated Pca, staging	5.5	Benign	PZ	3	4	Identified
15	13	Known Pca, assess change	4.16	3 + 4	PZ	4	3	Identified
16	14	No biopsy performed	7	NA	NA	2	2	Ignored
17	15	Benign	9.5	Benign	NA	1	2	Ignored

*NA, biopsies pathological score not available or cannot be determined; Identified, identified abnormality as stated; Additional, additional abnormality; Ignored, unable to locate abnormality*.

### Segmentation and Feature Extraction

Our study radiologists used MIM™ PACS [MIM Software Inc. (Cleveland, OH, USA)] to delineate regions of interest three-dimensionally (3D) on the prostate mpMRI scans, using T2w images as the reference sequence. Lesion boundaries were independently marked on the test and retest scans, whose cancer status was pathological identified by prior study. Four additional abnormalities that appeared radiologically malignant were identified and anatomically matched in sequential time points by our radiologists, but these lesions did not have pathological assessment. All lesion boundary segmentation was carried out as consensus reads by the study radiologists. Independent boundary delineation between lesions in the test and retest time point scan not only depicts the real clinical situation, but also introduces boundary variations, which can increase variability in the computed quantitative features.

Using the MIM libraries, T2w and ADC sequences were coregistered to avoid any motion artifacts in acquisition between the modalities. The registered multimodal image sequences were exported as 3D image matrices along with segmentation masks. We developed custom radiomic feature extraction tools, whose feature definition and formulation followed the IBSI consensus recommendations (25). We extracted 307 quantitative imaging features in the converged region of interest, which could be broadly categorized into three broad groups: C1: size and shape (45 features), C2: intensity, co-occurrence, run length (107 features), and C3: texture—laws and wavelets (155 features); see Supplemental Tables S1–S3.

### Standardization of Image Regions

To assess the role of standardization procedures on feature stability in test–retest imaging, we propose to use conventional *z*-score standardization. We started by segmenting the prostate gland in 3D, and the region voxels were standardized by subtracting the mean and dividing by the deviation obtained at the gland level. The standardization was carried out independently for each modality (T2w and ADC) at a patient level. The lesion region of interest is standardized at the scan level and tend to have relative intensity with respect to the entire gland for a patient scan.

### Habitat Image Region

We intend to find aggressive tumor-like regions in a marked lesion boundary of interest, which we call a *habitat* region. We define this region as one with restricted diffusion, whose characteristics resemble malignancy. We converge on a habitat region in two different ways: (a) sphere around lesion and (b) converge region within lesion. To find such a region, we first consider the entire lesion in 3D and on a colocalized volume across modalities. In the first case (a), we increase the search space to a 3D sphere with a fixed diameter of 15 mm and converge on a restricted diffusion region based on ADC values using a threshold defined by the distributional deviation (27, 28) and conforming regions to within the prostate gland structure. In the second case (b), we find the most contiguous lower median cutoff that is spanned in the ADC map within the radiologist-marked lesion region of interest. Converged habitat region will be mapped back to each modality of interest (T2w and ADC), and quantitative features are computed on a newly defined boundary. In the first case, it is possible to obtain a region larger than that marked by the radiologists. In the second case, the habitat region will always be contained within the marked lesion.

### Concordant Features

Quantitative features that are reproducible in repeated experiments and can describe differential physiology are a necessary step for consideration as biomarkers. The feature values that are consistent between the test and retest experiment were evaluated. For each image feature, the concordance correlation coefficient (CCC) was computed to quantify reproducibility between the two scans for a patient across the cohort and independently computed in each modality (T2w, ADC). The CCC measures deviation from the diagonal line averaged over samples in the cohort and is commonly used to measure fidelity in repeated experiments (25). On this set of highly reproducible features, the next step was to select the features with a large interpatient variability, measured using the *dynamic range* (DR) metric. The normalized DR for a feature was defined by the inverse of the ratio of the average difference between measurements to the observed interpatient variability or biological range:

(1) $$\begin{array}{r} \text{DR=}\left({\text{1-}}^{\text{1}}{/}_{n}\overset{n}{\underset{i\text{=1}}{\sum }}\frac{\left|\text{Test(}i\text{)-Retest(}i\text{)}\right|}{\text{Max-Min}}\right) \end{array}$$

where *n* is the total number of patient case; the DR varies from 0 to 1. Values close to 1 are preferred and imply that the feature has a large relative biological range, limited by the diversity in the cohort. As the variation between test–retest features increases, the DR values will show a reduction. Screening for a large DR will eliminate features that show greater variability in the repeat scans compared to the range of coverage. It is critical that a clinically relevant feature have a large DR to adequately distinguish the variations with tumor types, but show minimal variability in describing the same tumor type.

### Redundancy Reduction

We propose to eliminate redundancies in features that are found to be reproducible. We computed the coefficient of determination (*R*^2^) between the features that are considered to be reproducible, which measures the level of dependency between features. The *R*^2^ has a range of 0 to 1 and is a ratio of the known variance as measured by linear model to the total variance between two variables or features, where one is the outcome, and the other is used to form the predictor. Values close to 1 would mean that the data points are close to the fitted line (i.e., closer to dependency) (24, 25). The coefficient of determination of simple regression is equal to the square of the Pearson correlation coefficient (29, 30). The features were grouped based on the *R*^2^ values between them; in this subset, one representative was picked that had the highest DR. The procedure was repeated recursively to cover all the features. We implemented different cutoff values for *R*^2^ that assess linear dependence with any of the other features in the list. The purpose of this filter step is to eliminate redundancies, but not necessarily identify independence. The test–retest values were averaged before computing the *R*^2^. We set different cutoff limits to reduce redundancy and combine features that are over the cutoff range. We repeated this process for a range of cutoffs (0.95–0.99), in our study.

## Results

**Figure 2:**
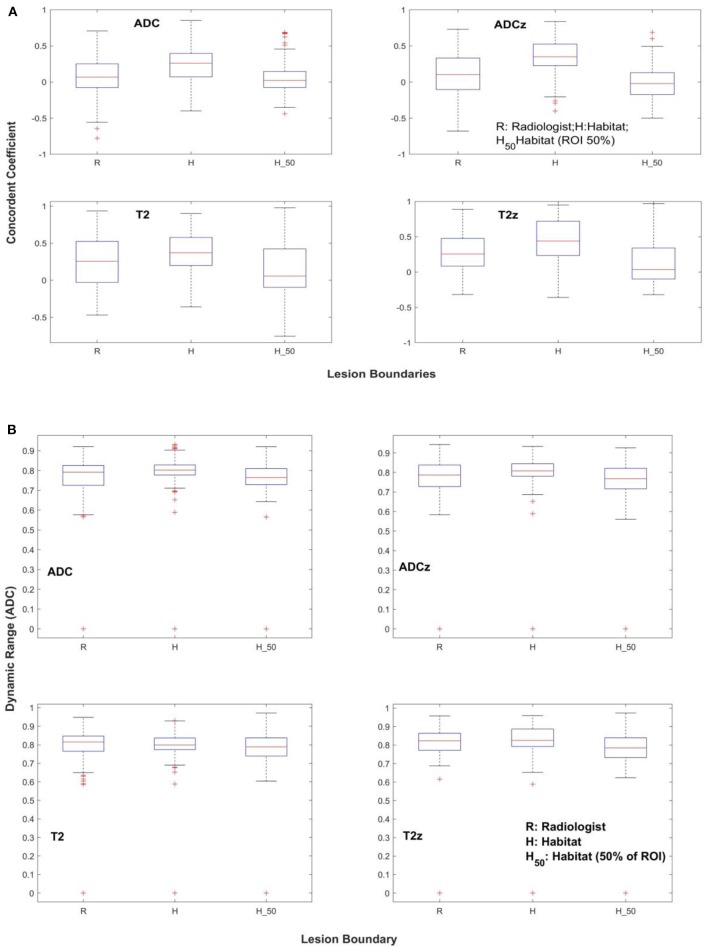
Repeatability of quantitative features across different lesion boundaries. **(A)** Concordance coefficient, **(B)** dynamic range.

As described in the *Materials and Methods*, the lesion was independently delineated in test and retest mpMRI scan, with each delineation done in consensus between the study radiologists. Using the lesion boundary as reference, the habitat region was converged automatically. We define habitat as a contiguous region colocalized to a low diffusion region defined by the ADC map. We then standardize the image voxels using *z*-score prior to any computations; in addition, we contrasted our findings with a non-standardized (raw) image region. In total, four image regions were investigated (raw-radiologist, *z*-score radiologist, raw-habitat, *z*-score habitat) by computing 307 quantitative image features in each of the regions, independently in test and retest images. We computed CCC to find repeatable image features, followed by application of a DR filter. Additionally, redundant features were removed based on coefficient of determination between the feature sets, repeated at different cutoffs. Distribution of features with different level settings in concordance correlation (CCC) and DR across the patient cohort is shown in Table 2. The imaging features that were extracted for respective modalities are listed in Table 3, obtained with *R*^2^ ≥ 0.99 (CCC and DR ≥0.65) and Supplemental Tables 4,5, obtained with *R*^2^ ≥ 0.95 (CCC and DR ≥0.65). Figure 2 shows the distribution of CCC and DR for features extracted using different boundary regions; radiologist delineated (R), habitat converged (H), and habitat within the manually delineated region (H_50_).

**Table 2:** Distribution of quantitative imaging features at various levels of concordance and redundancy limits (Rsq at ≥0.99 and ≥0.95) for regions identified by **(A)** radiologist marked and **(B)** habitats converged (sphere), **(C)** habitat converged (≤ median, ADC map).

**(A). Radiologist marked**				
**Concordance and dynamic range with redundancy reduction (Rsq** **≥** **0.99) Test–retest mpMRI: number of features (radiologist)**				
**CCC and DR and (Rsq** **≥** **0.99)**	**ADC**	**ADCz**	**T2**	**T2z**
≥0.95	0	0	0	0
≥0.90	0	0	3	0
≥0.85	0	0	4	1
≥0.80	0	0	5	3
≥0.75	0	0	9	6
≥0.70	1	3	13	10
≥0.65	2	3	19	12
**(B). Habitat converged (Sphere)**				
**Concordance and dynamic range with redundancy reduction (Rsq** **≥0.99) Test–retest mpMRI: number of features (habitats)**				
**CCC and DR and (Rsq** **≥0.95)**	**ADC**	**ADCz**	**T2**	**T2z**
≥0.95	0	0	0	1
≥0.90	0	0	1	2
≥0.85	1	0	3	6
≥0.80	4	3	6	11
≥0.75	6	5	8	14
≥0.70	9	8	13	18
≥0.65	15	15	18	23
**(C). Habitat (≤median ADC map)**				
**Concordance and dynamic range** **≥** **0.65 and Rsq** **≥0.99**				
**CCC and DR**	**ADC**	**ADCz**	**T2**	**T2z**
≥0.95	0	0	3	1
≥0.90	0	0	4	1
≥0.85	0	0	4	1
≥0.80	0	0	4	3
≥0.75	0	0	7	3
≥0.70	0	0	10	5
≥0.65	1	1	12	10

**Table 3:** Radiomic features that show concordance and non-redundancy in the test–retest cohort **(**CCC and DR ≥ 0.65;Rsq ≥ 0.99) for **(A)** radiologist-marked region, **(B)** habitat region (sphere, 15 mm), **(C)** habitat (≤ median, ADC).

**(A). Radiologist regions**	
**Radiologist marked region (T2): CCC and DR** **> = 0.65; Rsq** **≥** **0.99**.	
**T2 (raw): 19 features**	**T2 (*****z*****-normalized): 12 features**
F138:GLSZM_Large-zone-low-gray-level-emphasis- F149:NGTDM_Contrast- F10:Stat-Max-gray-level F150:NGTDM_Busyness- F107:avgCooc_3D_Inv-diff-mom-norm F284:3D-Wave-P1-L2-C4- F115:avgCooc_3D_Second-measure-of-information-correlation- F113:avgCooc_3D_Cluster-prominence-F93:avgCooc_3D_Joint-var F99:avgCooc_3D_Sum-var-F151:NGTDM_Complexity F302:3D-Wave-P1-L2-C13 F300:3D-Wave-P1-L2-C12 F27:Int-hist-90th-percentile F294:3D-Wave-P1-L2-C9 F304:3D-Wave-P1-L2-C14 F152:NGTDM_Strength	F19:Stat-Root-Mn-Sq- F149:NGTDM_Contrast- F10:Stat-Max-gray-level- F12:Stat-range- F107:avgCooc_3D_Inv-diff-mom-norm- F171:3D-LawsF-L5-R5-R5- F284:3D-Wave-P1-L2-C4- F3:Stat-SD- F152:NGTDM_Strength- F8:Stat-10th-percentile-
**Shape and Size**	**Shape and size**
F47:Vol-at-Int-fraction-diff F43:Vol-at-Int-Fraction-10-	F47:Vol-at-Int-fraction-diff- F43:Vol-at-Int-Fraction-10-
**Radiologist marked region (ADC):** CCC **and DR** **≥** **0.65; Rsq** **≥** **0.99**	
**ADC (raw): two features**	**ADC (*****z*****-normalized): three features**
F9:Stat-90th percentile F8:Stat-10th percentile	F96:avgCooc_3D_Difference-var F120:avg_3D_SRLGE-(Short-run-low-gray-level-emphasis) ***Shape and size*** F88:Center-of-mass-shift-(mm)
**(B). Habitat regions**	
**Habitat using sphere (ADC): CCC and DR** **≥** **0.65; Rsq** **≥** **0.99**	
**ADC (raw): 15 features**	**ADC (*****z*****-score): 15 features**
F228:3D-LawsF-R5-L5-L5-ADC-Auto F126:avg_3D_RLN-(Run-length-non-uniformity)-ADC F157:3D-LawsF-L5-L5-W5-ADC F231:3D-LawsF-R5-L5-R5-ADC F246:3D-LawsF-R5-R5-R5-ADC F170:3D-LawsF-L5-R5-S5-ADC F154:3D-LawsF-L5-L5-E5-ADC F155:3D-LawsF-L5-L5-S5-ADC F169:3D-LawsF-L5-R5-E5-ADC F171:3D-LawsF-L5-R5-R5-ADC F140:GLSZM_Gray-level-non-uniformity F124:avg_3D_GLN-(Gray-level-non-uniformity) F148:NGTDM_Coarseness	F302:3D-Wave-P1-L2-C13-ADC F294:3D-Wave-P1-L2-C9-ADC F218:3D-LawsF-S5-R5-L5-ADC F296:3D-Wave-P1-L2-C10-ADC F304:3D-Wave-P1-L2-C14-ADC F228:3D-LawsF-R5-L5-L5-ADC F243:3D-LawsF-R5-R5-L5-ADC F198:3D-LawsF-E5-W5-L5-ADC F151:NGTDM_Complexity-ADC F140:GLSZM_Gray-level-non-uniformity-ADC F7:Stat-Min-gray-level-ADC F115:avgCooc_3D_Second-measure-of-information-correlation-ADC F148:NGTDM_Coarseness-ADC
**Shape and size:**	**Shape and size:**
F90:Border-length-(mm) F54:Surface-area-(mm^∧^2)-ADC	F52:Vol-(mm^∧^3)-ADC F54:Surface-area-(mm^∧^2)-ADC
**Habitats using sphere (T2): CCC and DR** **> = 0.65; Rsq** **≥0.99**	
**T2 (raw): 18 features**	**T2 (*****z*****-normalized): 23 features**
F117:avg_3D_LRE-(Long-runs-emphasis)-ADC F126:avg_3D_RLN-(Run-length-non-uniformity)-ADC F144:GLSZM_Zone-percentage-ADC F27:Int-hist-90th-percentile-ADC F100:avgCooc_3D_Sum-entropy-ADC F161:3D-LawsF-L5-E5-R5-ADC F141:GLSZM_Gray-level-non-uniformity-normalized-ADC- F124:avg_3D_GLN-(Gray-level-non-uniformity)-ADC F150:NGTDM_Busyness-ADC F243:3D-LawsF-R5-R5-L5-ADC F149:NGTDM_Contrast-ADC F231:3D-LawsF-R5-L5-R5-ADC F300:3D-Wave-P1-L2-C12-ADC F140:GLSZM_Gray-level-non-uniformity-ADC F115:avgCooc_3D_Second-measure-of-information-correlation-ADC F114:avgCooc_3D_First-measure-of-information-correlation-ADC F148:NGTDM_Coarseness-ADC	F115:avgCooc_3D_Second-measure-of-information-correlation-ADC F149:NGTDM_Contrast-ADC F29:Int-hist-mode-ADC F19:Stat-Root-Mn-Sq-ADC F159:3D-LawsF-L5-E5-E5-ADC F122:avg_3D_LRLGE-(Long-run-low-gray-level-emphasis)-ADC F298:3D-Wave-P1-L2-C11-ADC F306:3D-Wave-P1-L2-C15-ADC F40:Max-hist-Gradient-gray-level-ADC F42:Min-hist-Gradient-gray-level-ADC F8:Stat-10th-percentile-ADC F136:GLSZM_Small-zone-low-gray-level-emphasis-ADC F152:NGTDM_Strength-ADC F139:GLSZM_Large-zone-high-gray-level-emphasis-ADC F97:avgCooc_3D_Difference-entropy-ADC- F41:Min-hist-Gradient-ADC- F140:GLSZM_Gray-level-non-uniformity-ADC F231:3D-LawsF-R5-L5-R5-ADC F54:Surface-area-(mm^∧^2)-ADC F114:avgCooc_3D_First-measure-of-information-correlation-ADC F148:NGTDM_Coarseness-ADC
**Shape and Size:**	**Shape and Size:**
F54:Surface-area-(mm^∧^2)-	F88:Center-of-mass-shift-(mm)-ADC F87:Weighted-CoM_z-(mm)-ADC
**(C). Habitat within lesion**	
**Habitat within lesion (≤** **Median): ADC: CCC and DR** **≥** **0.65; Rsq** **≥** **0.99**	
**ADC (raw): 1 feature**	**ADC (*****z*****-normalized): 1 feature**
F10:Stat-Max-gray-level-ADC	F143:GLSZM_Zone-size-non-uniformity-normalized-ADCz-Auto
**Habitat within lesion (≤** **Median): T2: CCC and DR** **≥** **0.65; Rsq** **≥0.99**	
**T2 (raw): 12 features**	**T2 (*****z*****-normalized): 10 features**
F93:avgCooc_3D_Joint-var-ADC-T2 F151:NGTDM_Complexity-ADC-T2 F152:NGTDM_Strength-ADC-T2 F27:Int-hist-90th-percentile-ADC-T2 F131:avg_3D_RE-(Run-entropy)-ADC-T2 F113:avgCooc_3D_Cluster-prominence-ADC-T2 F282:3D-Wave-P1-L2-C3-ADC-T2 F290:3D-Wave-P1-L2-C7-ADC-T2 F149:NGTDM_Contrast-ADC-T2 F18:Stat-ENERGY-ADC-T2 F40:Max-hist-Gradient-gray -level-ADC-T2 F99:avgCooc_3D_Sum-var-ADC-T2	F3:Stat-SD-T2z F9:Stat-90th-percentile-T2z F149:NGTDM_Contrast-T2z F93:avgCooc_3D_Joint-var-T2z F27:Int-hist-90th-percentile-T2z F113:avgCooc_3D_Cluster-prominence-T2z F151:NGTDM_Complexity-T2z F152:NGTDM_Strength-T2z F19:Stat-Root-Mn-Sq-T2z F30:Int-hist-interquartile-range-T2z

In our analysis, we find there are similar distributions of features between T2w- raw (native intensity values), radiologist-marked regions (19 features, CCC ≥0.65), and T2w-habitat with sphere regions (18 features, CCC ≥0.65), with standardized T2w *z*-score habitat (23 features, CCC ≥0.65) regions showing more stable features compared to T2w *z*-score raw regions. There were 12 stable features in T2w and 10 in T2w *z*-normalized regions, both evaluated at CCC ≥0.65 and with redundancy *R*^2^ ≥ 0.99 (Tables 2B,C, 3B,C). Of the 19 features that are stable in T2w radiologist-marked regions, there are two volume features that measure within a certain intensity range and 17 others that are texture features.

In ADC map images, there were two features found within radiologist-marked regions compared to three features in ADC *z*-score regions, both evaluated at CCC ≥0.65 and with redundancy *R*^2^ ≥ 0.99. Using ADC-sphere–based habitats, we find the number of stable features increased to 15 in the ADC-habitat, seen in both radiologist-marked and *z*-scored normalized regions. While using habitat region within lesion approach, the new region was restricted to be within the lesion. We find there was one stable feature in ADC and ADC *z*-normalized region; in these regions, five and one feature were concordant, respectively (see Supplemental Tables). It seems *z*-score standardization moderately helps to improve the number of repeatable features in ADC maps.

**Figure 3:**
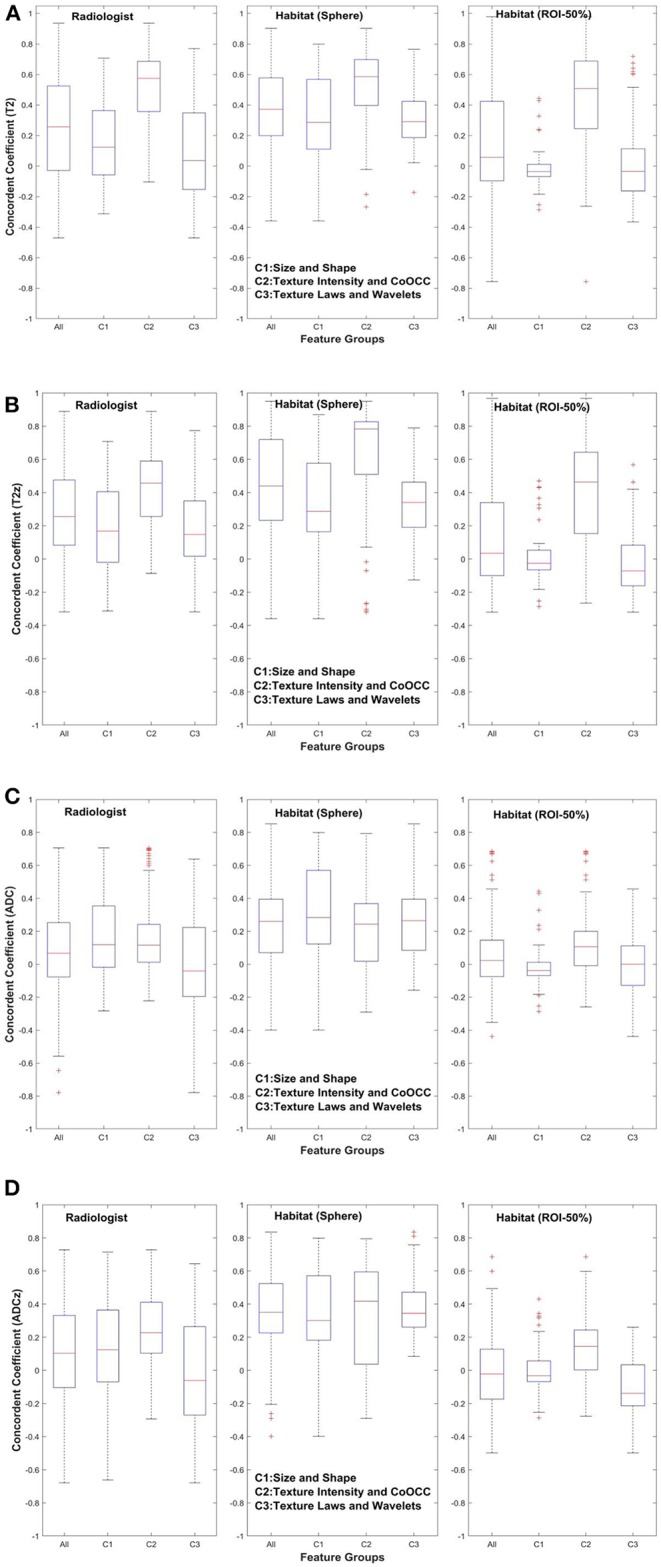
Concordance of quantitative features across feature subgroup. **(A)** T2w, **(B)** T2w *z*-normalized, **(C)** ADC, **(D)** ADC *z*-normalized.

**Figure 4:**
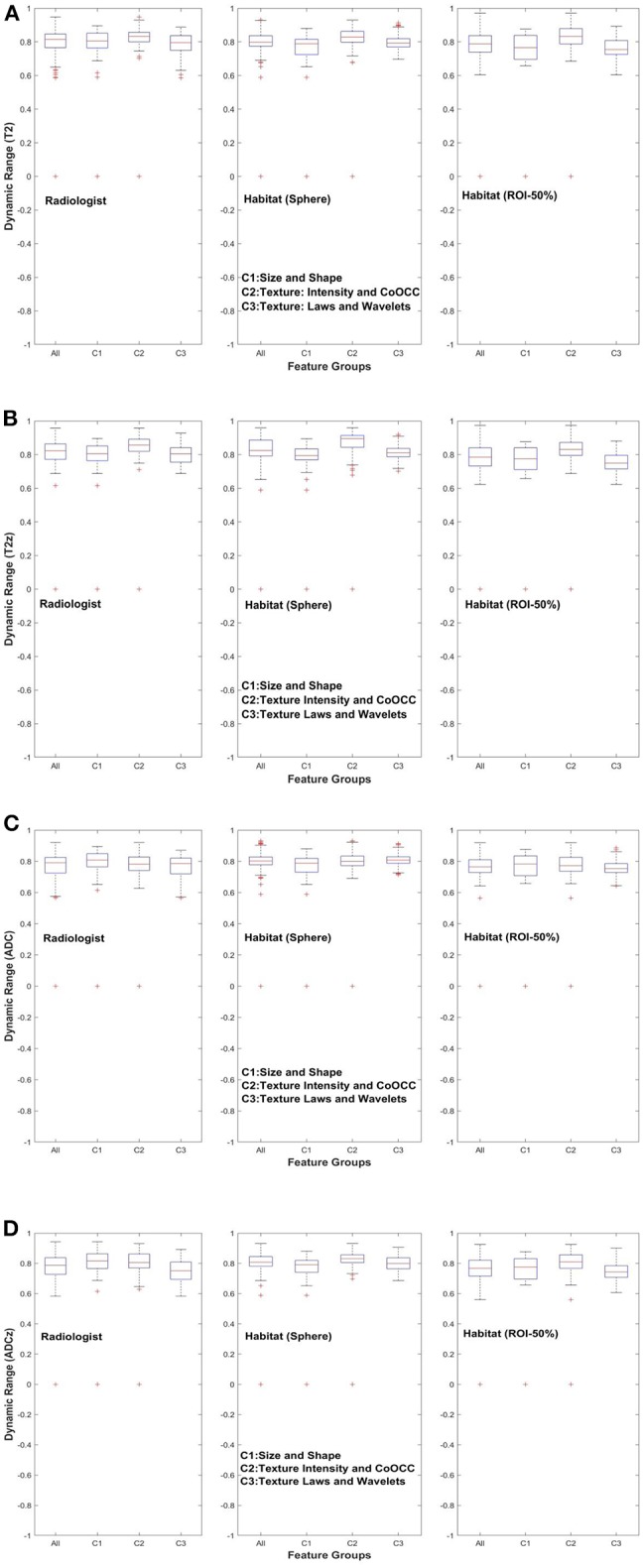
Dynamic range of quantitative features across feature subgroup. **(A)** T2w, **(B)** T2w *z*-normalized, **(C)** ADC, **(D)** ADC *z*-normalized.

Figures 3, 4 show the distribution of concordance coefficient and DR metric values, computed on features, respectively. They are grouped into the following broad categories: size and shape (C1), intensity and co-occurrence (C2), and laws and wavelets (C3). Texture features in the C2 intensity and co-occurrence category show higher concordance compared to other categories of features in T2w. The features computed in ADC map do not show any consistent trend. It is also interesting to note that features in size and shape categories show lower concordance values.

While the ADC map shows intensity statistics to be reproducible, the *z*-score region shows reproducible co-occurrence matrix. The habitat region using sphere approach shows more than eight features related to fine texture (Laws) and two features related to shape category. While the T2-habitat (sphere) shows more features from co-occurrence, neighborhood gray tone difference categories. In region converged by habitat within lesions, ADC map shows one feature related to gray level that is stable and non-redundant. The T2-habitat (within lesion) shows 12 features that are related to texture–neighborhood gray tone, co-occurrence, and wavelet based. The *z*-score standardization in T2-habitat (within lesion) region shows features related to gray-level intensities, co-occurrence, and neighborhood gray tone features that are reproducible and stable.

## Discussion

Clinically relevant imaging biomarkers are expected to be repeatable in a test–retest patient cohort, reproducible across centers, and relevant to describing the tumor physiology across different conditions. It is essential for imaging features to be used as a biomarker to be repeatable, at an acceptable level, which is dependent on the current imaging technology. In our study, we obtained prostate patient mpMRI scans within 2 weeks of the baseline time point and believe that the cohort is a unique public data set in prostate cancer. While we understand that the cohort size may be small for obtaining elaborate inferences, the methods applied by our study nonetheless allow us to assess feature stability and generate potential biomarkers in prostate mpMRI. We analyzed the repeatability in four different regions: (a) raw, radiologist-drawn; (b) *Z* score, radiologist drawn; (c) raw, habitats; (d) *z*-score, habitat regions. The study allowed us to contrast the reproducible features under these constraints.

The sphere-based habitat tends to increase the capture region that may provide a larger lesion boundary. This certainly helps to find stable and reproducible features in the ADC map and T2w image region. In comparison to habitat region formed within the lesion, it seems to restrict ADC intensity gray level that helps to find stable features in T2w, with more than 21 features with high concordance (CCC ≥0.75), of which 18 features are stable and reproducible (CCC ≥0.85, *R*^2^ > 0.099).

We believe that the habitat approach reduces variability in T2w and rather highly variable ADC map images, which typically have lower resolution. There are a number of automated and semiautomated segmentation procedures that could be used in mpMRI, but we restricted our approach to manual, expert radiologist–drawn boundaries to initially delineate the lesions. We used the manual segmented region as an initial seed point for habitat region delineation, which is automatically converged using multimodal sequences (T2w, ADC).

In a prior study (23), they used an interclass correlation with a cutoff of 0.85 and reported features related to entropy, inverse difference moments to be highly repeatable. In our study, we find that co-occurrence and neighborhood gray tone difference matrices (NGTDMs) are two feature categories that are repeatable in T2w and T2w z. In ADC maps, the statistics of intensity-type features seem to show up as stable even in raw intensities (without any standardization), whereas average co-occurrence, short run length gray level emphasis-type features are stable in *z*-normalized ADC maps. We also find habitat (sphere) approach seems to improve the number of repeatable features in ADC maps and in T2w (Figure 2).

In the previously mentioned study, the authors claimed neither standardization nor prefiltering improved repeatability of image features. In our study, we used CCC with additional criteria such as DR and redundancy reduction to filter the features. We also find that most size and shape–based features show lower concordance in T2w/T2w *z*, but a larger spread on ADC map in comparison to two categories of features (Figure 3). This is probably due to the use of different regional convergence methods coupled with independently defined, delineated lesion boundaries in the test and retest scans. In comparison, the prior study (23) claimed high concordance for features in the size and shape–based category.

Because of scan quality limitations, some of the prior marked regions could not be ascertained by our radiologist, and additional regions of abnormality were located in consensus by the study radiologists. Additionally, prior studies (22, 23) restricted lesions to the peripheral zone, while our study radiologists identified lesions without any zonal restrictions. These differences have certainly increased the feature variability, which could be one cause for a lower number of repeatable features. Nevertheless, our cohort of patients provides a diverse set of lesions that are spread across the gland. The habitat approach proposed in the study shows promise in increasing the number of repeatable imaging features.

### Study Limitations

This study provides a unique patient cohort with test–retest scans obtained within 2 weeks between scans; the cohort size is certainly a limited factor for a broader inference. The methodology used in the study with endorectal coils introduced artifacts that could have altered the voxel intensities and influenced the image feature reproducibility. We have taken effort to remove patient scans that show large artifacts and regions that could not be converged in a consensus read. Despite our efforts, there could be a certain level of variation in features value due to voxel level changes.

## Conclusions

In the current study, we demonstrate that there are quantitative imaging features that can be obtained repeatedly in prostate mpMRI. We show that sublocalized regions or habitats can improve repeatability of imaging features, possibly by restricting the range of variations in the voxel intensity levels in these MRI scan modalities. We also find that *z*-score normalization of the image intensities had minimal effect on the feature reproducibility. Current findings allow us to obtain reproducible and non-redundant sets of image features that could be used for predictive and prognostic purpose.

## Data Availability Statement

Dataset used in this study can be accessed using following URL: https://wiki.cancerimagingarchive.net/display/Public/QIN-PROSTATE-Repeatability and additional analysis, study information for this study are included in the article/Supplementary Material.

## Ethics Statement

The studies involving human participants were reviewed and approved by USF IRB. The ethics committee waived the requirement of written informed consent for participation.

## Author Contributions

HL and YB: Hypothesis, methods development. HL, JQ, QL, KG, and SFa: lesions identification and marking. YB, NP, and HL: results inference, implementation of methods. YB, HL, and JC: manuscript writing. HL, YB, RG, JP-S, JC, JQ, QL, KG, SFa, and SFe: manuscript/results proof read and approval.

## Conflict of Interest

HL, QL, and SFa received research scholarship to partial support their salary from Tianjin Medical and Cancer Hospital, Tianjin, China during their tenured research time at Moffitt Cancer Center. RG is an investor and consultant in Health Myne. The remaining authors declare that the research was conducted in the absence of any commercial or financial relationships that could be construed as a potential conflict of interest.
