# Supplementary material for: Repeatability of Quantitative Imaging Features in Prostate Magnetic Resonance Imaging
Source: Front Oncol. 2020 May 7;10:551. doi: 10.3389/fonc.2020.00551 (PMC7221156; doi:10.3389/fonc.2020.00551)
Supplement: Supplementary file 1 [file Data_Sheet_1.PDF]

SUPPLEMENTAL SECTION for

Repeatability of Quantitative Imaging Features in Prostate Magnetic Resonance Imaging

H. Lu[1,2], N. A. Parra[2], J. Qi[2], K. Gage[3], Qian Li[1], Shuxuan Fan[1,2], S. Feuerlein[3], J. Pow-Sang[4], R. Gillies[2,3], J. W. Choi[3], Y.Balagurunathan[3,4,5]

[1]Tianjin Medical and Cancer Hospital, Tianjin, China
Department of [2]Cancer Physiology, [3]Diagnostic Imaging, [4]Genitourinary Oncology, [5]Bioinformatics & Biostatistics, Moffitt Cancer Center, Tampa, FL.

Corresponding author
Jung Choi, M.D, Ph.D & Yoganand Balagurunthan, Ph.D.
Department of Biostatistics & Bioinformatics,
H. L. Moffitt Cancer Center,
12902 USF. Magnolia Ave,
Tampa, FL 33612.
Email: {jung.choi or yogab}@moffitt.org

Supplemental Table S.1. Feature categories (3D Image features).

**Category**	**Description**	**Number of Descriptors**
C1: Tumor Size & Shape	Size, location, volume descriptors	45
C2: Pixel Intensity Histogram, Grayscale: Runlength & CoOccurrence	Pixel intensity histogram statistics, Run length and Co-occurrence patterns.	107
C3: Texture: Laws & Wavelets	Laws Kernel (energy) and Wavelet kernels (entropy and energy)	155
	**Total**	**307**

Supplemental Table S.2. Quantitative Image features used to describe the lesion of interest, indepdently in each

Sno	Feature Index	Description of the Features	Feature Category
1	F43	Vol-at-Int-Fraction-10-T2	**C1: Tumor Size & Shape**
2	F44	Vol-at-Int-fraction-90-T2	
3	F47	Vol-at-Int-fraction-diff-T2	
4	F49	Area-under-IVH-curve-T2	
5	F50	Num-of-connect-3D-comp-T2	
6	F51	Longest-diameter(mm)-T2	
7	F52	Vol-(mm^3)-T2	
8	F53	Vol-(pxl)-T2	
9	F54	Surface-area-(mm^2)-T2	
10	F55	Surface-to-Vol-ratio-(mm^2)-T2	
11	F56	Compactness-1-T2	
12	F57	Compactness-2-T2	
13	F58	Spherical-disproportion-T2	
14	F59	Sphericity-T2	
15	F60	Asphericity-T2	
16	F61	Major-axis-length-T2	
17	F62	Minor-axis-length-T2	
18	F63	Least-axis-length-T2	
19	F64	Elongation-T2	
20	F65	Flatness-T2	
21	F66	Vol-density--axis-aligned-bounding-box-T2	
22	F67	Area-density--axis-aligned-bounding-box-T2	
23	F68	Vol-density--oriented-bounding-box-T2	
24	F69	Area-density--oriented-bounding-box-T2	
25	F70	Vol-density--approximate-enclosing-ellipsoid-T2	
26	F71	Area-density--approximate-enclosing-ellipsoid-T2	
27	F72	Vol-density--Min-Vol-enclosing-ellipsoid-T2	
28	F73	Area-density--Min-Vol-enclosing-ellipsoid-T2	
29	F74	Vol-density--convex-hull-T2	
30	F75	Area-density--convex-hull-T2	
31	F76	Asymmetry-T2	
32	F77	Eccentricity-T2	
33	F78	Orientation-T2	
34	F79	CoM_x-(pxl)-T2	
35	F80	CoM_y-(pxl)-T2	
36	F81	CoM_z-(pxl)-T2	
37	F82	CoM_x-(mm)-T2	
38	F83	CoM_y-(mm)-T2	
39	F84	CoM_z-(mm)-T2	
40	F85	Weighted-CoM_x-(mm)-T2	
41	F86	Weighted-CoM_y-(mm)-T2	
42	F87	Weighted-CoM_z-(mm)-T2	

43	F88	Centre-of-mass-shift-(mm)-T2	
44	F89	Border-length-(pxl)-T2	
45	F90	Border-length-(mm)-T2	
46	F1	Stat-Mean-T2	**C2: Pixel Intensity Histogram, Grayscale**: Runlength & CoOccurrence
47	F2	Stat-Variance-T2	
48	F3	Stat-SD-T2	
49	F4	Stat-SKEW-T2	
50	F5	Stat-Kurtosis-T2	
51	F6	Stat-Median-T2	
52	F7	Stat-Min-grey-level-T2	
53	F8	Stat-10th-percentile-T2	
54	F9	Stat-90th-percentile-T2	
55	F10	Stat-Max-grey-level-T2	
56	F11	Stat-Interquartile-range-T2	
57	F12	Stat-range-T2	
58	F13	Stat-Mean-Abs-Dev-T2	
59	F14	Stat-Robust-Mean-Abs-Dev-T2	
60	F15	Stat-Med-Abs-Dev-T2	
61	F16	Stat-Coef-of-var-T2	
62	F17	Stat-Quart-coef-of-dispr-T2	
63	F18	Stat-ENERGY-T2	
64	F19	Stat-Root-Mn-Sq-T2	
59	F20	Int-hist-Mn-T2	
60	F21	Int-hist-Var-T2	
61	F22	Int-hist-Skew-T2	
62	F23	Int-hist-Kurtosis-T2	
63	F24	Int-hist-Med-T2	
64	F25	Int-hist-Min-grey-level-T2	
65	F26	Int-hist-10th-Percentile-T2	
66	F27	Int-hist-90th-percentile-T2	
67	F28	Int-hist-Max-grey-level-T2	
68	F29	Int-hist-mode-T2	
69	F30	Int-hist-interquartile-range-T2	
70	F31	Int-hist-range-T2	
71	F32	Int-hist-Mean-Abs-Dev-T2	
72	F33	Int-hist-Robust-Mn-Abs-Dev-T2	
73	F34	Int-hist-Med-Abs-Dev-T2	
74	F35	Int-hist-Coef-of-Var-T2	
76	F36	Int-hist-Quart-Coeff-of-Dispr-T2	
77	F37	Int-hist-Entropy-T2	
78	F38	Int-hist-Uniformity-T2	
79	F39	Max-hist-Gradient-T2	
79	F40	Max-hist-Gradient-grey-level-T2	
80	F41	Min-hist-Gradient-T2	
81	F42	Min-hist-Gradient-grey-level-T2	
82	F45	Int-at-Vol-fraction-10-T2	
83	F46	Int-at-Vol-fraction-90-T2	

84	F48	Int-at-Vol-fraction-diff-T2	
85	F91	avgCooc_3D_Joint-MAX-T2	
86	F92	avgCooc_3D_Joint-Average-T2	
87	F93	avgCooc_3D_Joint-variance-T2	
88	F94	avgCooc_3D_Joint-entropy-T2	
89	F95	avgCooc_3D_Difference-average-T2	
90	F96	avgCooc_3D_Difference-variance-T2	
91	F97	avgCooc_3D_Difference-entropy-T2	
92	F98	avgCooc_3D_Sum-average-T2	
93	F99	avgCooc_3D_Sum-variance-T2	
94	F100	avgCooc_3D_Sum-entropy-T2	
95	F101	avgCooc_3D_Angular-second-moment-T2	
96	F102	avgCooc_3D_Contrast-T2	
97	F103	avgCooc_3D_Dissimilarity-T2	
98	F104	avgCooc_3D_Inv-diff-(Homogeneity)-T2	
99	F105	avgCooc_3D_Inv-diff-norm-(Homogeneity-normalized)-T2	
100	F106	avgCooc_3D_Inv-diff-mom-T2	
101	F107	avgCooc_3D_Inv-diff-mom-norm-T2	
102	F108	avgCooc_3D_Inv-variance-T2	
103	F109	avgCooc_3D_Correlation-T2	
104	F110	avgCooc_3D_Autocorrelation-T2	
105	F111	avgCooc_3D_Cluster-tendency-T2	
106	F112	avgCooc_3D_Cluster-shade-T2	
107	F113	avgCooc_3D_Cluster-prominence-T2	
108	F114	avgCooc_3D_First-measure-of-information-correlation-T2	
109	F115	avgCooc_3D_Second-measure-of-information-correlation-T2	
110	F116	avg_3D_SRE-(Short-runs-emphasis)-T2	
111	F117	avg_3D_LRE-(Long-runs-emphasis)-T2	
112	F118	avg_3D_LGRE-(Low-grey-level-run-emphasis)-T2	
113	F119	avg_3D_HGRE-(High-grey-level-run-emphasis)-T2	
114	F120	avg_3D_SRLGE-(Short-run-low-grey-level-emphasis)-T2	
115	F121	avg_3D_SRHGE-(Short-run-high-grey-level-emphasis)-T2	
116	F122	avg_3D_LRLGE-(Long-run-low-grey-level-emphasis)-T2	
117	F123	avg_3D_LRHGE-(Long-run-high-grey-level-emphasis)-T2	
118	F124	avg_3D_GLN-(Grey-level-non-uniformity)-T2	
119	F125	avg_3D_GLN-normalize-(Grey-level-non-uniformity-normalised)-T2	
120	F126	avg_3D_RLN-(Run-length-non-uniformity)-T2	
121	F127	avg_3D_RLN-normalize-(Run-length-non-	

		uniformity-normalised)-T2	
122	F128	avg_3D_RP-(Run-percentage)-T2	
123	F129	avg_3D_GV-(Grey-level-variance)-T2	
124	F130	avg_3D_RLV-(Run-length-variance)-T2	
125	F131	avg_3D_RE-(Run-entropy)-T2	
126	F132	GLSZM_Small-zone-emphasis-T2	
127	F133	GLSZM_Large-zone-emphasis-T2	
128	F134	GLSZM_Low-grey-level-zone-emphasis-T2	
129	F135	GLSZM_High-grey-level-zone-emphasis-T2	
130	F136	GLSZM_Small-zone-low-grey-level-emphasis-T2	
131	F137	GLSZM_Small-zone-high-grey-level-emphasis-T2	
132	F138	GLSZM_Large-zone-low-grey-level-emphasis-T2	
133	F139	GLSZM_Large-zone-high-grey-level-emphasis-T2	
134	F140	GLSZM_Grey-level-non-uniformity-T2	
135	F141	GLSZM_Grey-level-non-uniformity-normalised-T2	
136	F142	GLSZM_Zone-size-non-uniformity-T2	
137	F143	GLSZM_Zone-size-non-uniformity-normalised-T2	
138	F144	GLSZM_Zone-percentage-T2	
139	F145	GLSZM_Grey-level-variance-T2	
140	F146	GLSZM_Zone-size-variance-T2	
141	F147	GLSZM_Zone-size-entropy-T2	
142	F148	NGTDM_Coarseness-T2	
143	F149	NGTDM_Contrast-T2	
144	F150	NGTDM_Busyness-T2	
145	F151	NGTDM_Complexity-T2	
146	F152	NGTDM_Strength-T2	
157	F153	3D-LawsF-L5-L5-L5-T2	**C3. Laws & Wavelet Texture** (feature at different layers)
158	F154	3D-LawsF-L5-L5-E5-T2	
159	F155	3D-LawsF-L5-L5-S5-T2	
160	F156	3D-LawsF-L5-L5-R5-T2	
161	F157	3D-LawsF-L5-L5-W5-T2	
162	F158	3D-LawsF-L5-E5-L5-T2	
163	F159	3D-LawsF-L5-E5-E5-T2	
164	F160	3D-LawsF-L5-E5-S5-T2	
165	F161	3D-LawsF-L5-E5-R5-T2	
166	F162	3D-LawsF-L5-E5-W5-T2	
167	F163	3D-LawsF-L5-S5-L5-T2	
168	F164	3D-LawsF-L5-S5-E5-T2	
169	F165	3D-LawsF-L5-S5-S5-T2	
170	F166	3D-LawsF-L5-S5-R5-T2	
171	F167	3D-LawsF-L5-S5-W5-T2	
172	F168	3D-LawsF-L5-R5-L5-T2	

173	F169	3D-LawsF-L5-R5-E5-T2	
174	F170	3D-LawsF-L5-R5-S5-T2	
175	F171	3D-LawsF-L5-R5-R5-T2	
176	F172	3D-LawsF-L5-R5-W5-T2	
177	F173	3D-LawsF-L5-W5-L5-T2	
178	F174	3D-LawsF-L5-W5-E5-T2	
179	F175	3D-LawsF-L5-W5-S5-T2	
180	F176	3D-LawsF-L5-W5-R5-T2	
181	F177	3D-LawsF-L5-W5-W5-T2	
182	F178	3D-LawsF-E5-L5-L5-T2	
183	F179	3D-LawsF-E5-L5-E5-T2	
	F180	3D-LawsF-E5-L5-S5-T2	
190	F181	3D-LawsF-E5-L5-R5-T2	
191	F182	3D-LawsF-E5-L5-W5-T2	
192	F183	3D-LawsF-E5-E5-L5-T2	
193	F184	3D-LawsF-E5-E5-E5-T2	
194	F185	3D-LawsF-E5-E5-S5-T2	
195	F186	3D-LawsF-E5-E5-R5-T2	
196	F187	3D-LawsF-E5-E5-W5-T2	
197	F188	3D-LawsF-E5-S5-L5-T2	
198	F189	3D-LawsF-E5-S5-E5-T2	
199	F190	3D-LawsF-E5-S5-S5-T2	
200	F191	3D-LawsF-E5-S5-R5-T2	
201	F192	3D-LawsF-E5-S5-W5-T2	
202	F193	3D-LawsF-E5-R5-L5-T2	
203	F194	3D-LawsF-E5-R5-E5-T2	
204	F195	3D-LawsF-E5-R5-S5-T2	
205	F196	3D-LawsF-E5-R5-R5-T2	
206	F197	3D-LawsF-E5-R5-W5-T2	
207	F198	3D-LawsF-E5-W5-L5-T2	
208	F199	3D-LawsF-E5-W5-E5-T2	
209	F200	3D-LawsF-E5-W5-S5-T2	
210	F201	3D-LawsF-E5-W5-R5-T2	
211	F202	3D-LawsF-E5-W5-W5-T2	
212	F203	3D-LawsF-S5-L5-L5-T2	
213	F204	3D-LawsF-S5-L5-E5-T2	
214	F205	3D-LawsF-S5-L5-S5-T2	
215	F206	3D-LawsF-S5-L5-R5-T2	
216	F207	3D-LawsF-S5-L5-W5-T2	
217	F208	3D-LawsF-S5-E5-L5-T2	
218	F209	3D-LawsF-S5-E5-E5-T2	
219	F210	3D-LawsF-S5-E5-S5-T2	
220	F211	3D-LawsF-S5-E5-R5-T2	
221	F212	3D-LawsF-S5-E5-W5-T2	
222	F213	3D-LawsF-S5-S5-L5-T2	
223	F214	3D-LawsF-S5-S5-E5-T2	
224	F215	3D-LawsF-S5-S5-S5-T2	
225	F216	3D-LawsF-S5-S5-R5-T2	
226	F217	3D-LawsF-S5-S5-W5-T2	

227	F218	3D-LawsF-S5-R5-L5-T2	
228	F219	3D-LawsF-S5-R5-E5-T2	
229	F220	3D-LawsF-S5-R5-S5-T2	
230	F221	3D-LawsF-S5-R5-R5-T2	
231	F222	3D-LawsF-S5-R5-W5-T2	
232	F223	3D-LawsF-S5-W5-L5-T2	
233	F224	3D-LawsF-S5-W5-E5-T2	
234	F225	3D-LawsF-S5-W5-S5-T2	
235	F226	3D-LawsF-S5-W5-R5-T2	
236	F227	3D-LawsF-S5-W5-W5-T2	
237	F228	3D-LawsF-R5-L5-L5-T2	
238	F229	3D-LawsF-R5-L5-E5-T2	
239	F230	3D-LawsF-R5-L5-S5-T2	
240	F231	3D-LawsF-R5-L5-R5-T2	
241	F232	3D-LawsF-R5-L5-W5-T2	
242	F233	3D-LawsF-R5-E5-L5-T2	
243	F234	3D-LawsF-R5-E5-E5-T2	
244	F235	3D-LawsF-R5-E5-S5-T2	
245	F236	3D-LawsF-R5-E5-R5-T2	
246	F237	3D-LawsF-R5-E5-W5-T2	
247	F238	3D-LawsF-R5-S5-L5-T2	
248	F239	3D-LawsF-R5-S5-E5-T2	
249	F240	3D-LawsF-R5-S5-S5-T2	
250	F241	3D-LawsF-R5-S5-R5-T2	
251	F242	3D-LawsF-R5-S5-W5-T2	
252	F243	3D-LawsF-R5-R5-L5-T2	
253	F244	3D-LawsF-R5-R5-E5-T2	
254	F245	3D-LawsF-R5-R5-S5-T2	
255	F246	3D-LawsF-R5-R5-R5-T2	
256	F247	3D-LawsF-R5-R5-W5-T2	
257	F248	3D-LawsF-R5-W5-L5-T2	
258	F249	3D-LawsF-R5-W5-E5-T2	
259	F250	3D-LawsF-R5-W5-S5-T2	
260	F251	3D-LawsF-R5-W5-R5-T2	
261	F252	3D-LawsF-R5-W5-W5-T2	
262	F253	3D-LawsF-W5-L5-L5-T2	
263	F254	3D-LawsF-W5-L5-E5-T2	
264	F255	3D-LawsF-W5-L5-S5-T2	
265	F256	3D-LawsF-W5-L5-R5-T2	
266	F257	3D-LawsF-W5-L5-W5-T2	
267	F258	3D-LawsF-W5-E5-L5-T2	
268	F259	3D-LawsF-W5-E5-E5-T2	
269	F260	3D-LawsF-W5-E5-S5-T2	
270	F261	3D-LawsF-W5-E5-R5-T2	
271	F262	3D-LawsF-W5-E5-W5-T2	
272	F263	3D-LawsF-W5-S5-L5-T2	
273	F264	3D-LawsF-W5-S5-E5-T2	
274	F265	3D-LawsF-W5-S5-S5-T2	
275	F266	3D-LawsF-W5-S5-R5-T2	

276	F267	3D-LawsF-W5-S5-W5-T2	
277	F268	3D-LawsF-W5-R5-L5-T2	
278	F269	3D-LawsF-W5-R5-E5-T2	
279	F270	3D-LawsF-W5-R5-S5-T2	
280	F271	3D-LawsF-W5-R5-R5-T2	
281	F272	3D-LawsF-W5-R5-W5-T2	
282	F273	3D-LawsF-W5-W5-L5-T2	
283	F274	3D-LawsF-W5-W5-E5-T2	
284	F275	3D-LawsF-W5-W5-S5-T2	
285	F276	3D-LawsF-W5-W5-R5-T2	
286	F277	3D-LawsF-W5-W5-W5-T2	
287	F278	3D-Wave-P1-L2-C1-T2	
288	F279	3D-Wave-P2-L2-C1-T2	
289	F280	3D-Wave-P1-L2-C2-T2	
290	F281	3D-Wave-P2-L2-C2-T2	
291	F282	3D-Wave-P1-L2-C3-T2	
292	F283	3D-Wave-P2-L2-C3-T2	
293	F284	3D-Wave-P1-L2-C4-T2	
294	F285	3D-Wave-P2-L2-C4-T2	
295	F286	3D-Wave-P1-L2-C5-T2	
296	F287	3D-Wave-P2-L2-C5-T2	
297	F288	3D-Wave-P1-L2-C6-T2	
298	F289	3D-Wave-P2-L2-C6-T2	
299	F290	3D-Wave-P1-L2-C7-T2	
300	F291	3D-Wave-P2-L2-C7-T2	
301	F292	3D-Wave-P1-L2-C8-T2	
302	F293	3D-Wave-P2-L2-C8-T2	
303	F294	3D-Wave-P1-L2-C9-T2	
304	F295	3D-Wave-P2-L2-C9-T2	
305	F296	3D-Wave-P1-L2-C10-T2	
306	F297	3D-Wave-P2-L2-C10-T2	
307	F298	3D-Wave-P1-L2-C11-T2	

Supplemental Table S3. Description of Texture Features

A. **Run-length analysis:** Run-length texture features (1) examine runs of similar gray values in an image. Runs may be labeled according to their length, gray value, and direction (either horizontal or vertical). Long runs of the same gray value correspond to coarser textures, whereas shorter runs correspond to finer textures. In our study, texture information was quantified by computing 11 features (2) derived from the run-length distribution matrix. They are: 1: Short Run Emphasis (SRE). 2: Long Run Emphasis (LRE). 3: Gray-Level Non-uniformity (GLN). 4: Run Length Non-uniformity (RLN). 5: Run Percentage (RP). 6: Low Gray-Level Run Emphasis (LGRE). 7: High Gray-Level Run Emphasis (HGRE). 8: Short Run Low Gray-Level Emphasis (SRLGE). 9: Short Run High Gray-Level Emphasis (SRHGE). 10: Long Run Low Gray-Level Emphasis (LRLGE). 11: Long Run High Gray-Level Emphasis (LGHGE).

   The Co-occurrence matrices and run-length analysis features can be obtained in 3D (3), the features are calculated in 13 different directions, in each direction, processing is done by plane instead of slice. Hence, information between slices is used.

B. **Co-occurrence matrices:** the co-occurrence matrix (4) is a matrix that contains the frequency of one gray level intensity appearing in a specified spatial linear relationship with another gray level intensity within a certain range. Computation of features requires first constructing the co-occurrence matrix, then different measurements (5) can be calculated based on the matrix. The measurements include: contrast, energy, homogeneity, entropy, mean and max probability.

C. **Laws features :** Laws features (6) were constructed from a set of five one-dimensional filters, each designed to reflect a different type of structure in the image. These one-dimensional filters are defined as E5 (edges), S5 (spots), R5 (ripples), W5 (waves), and L5 (low pass, or average gray value). By using these 1-D convolution filters, 2-D filters are generated by convolving pairs of these filters, such as L5L5, E5L5, S5L5, W5L5, R5L5, etc. We can generate 25 different 2-D filters. 3D laws filters were constructed similarly to 2D. 3D filters are generated by convolving 3 types of 1D filter, such as L5L5L5, L5L5E5, L5L5S5, L5L5R5, L5L5W5, etc. The total number of 3-D filters is 125. For the 3D case, after the convolution with the 3D filters for the image, the energy (7) of the texture feature was computed by the following equation:

$$Energy = \frac{1}{R}\sum_{i=N+1}^{I-N}\sum_{j=N+1}^{J-N}\sum_{k=N+1}^{K-N}h^2(i,j,k)$$,

   where R is a normalizing factor, I and J, K are image dimensions, h(i,j,k) is derived from the convolution filters and original image. For the 2D case, the above equation is very similar, but without the 3rd (z direction) dimension.

D. **Wavelet Decomposition:**
   The discrete wavelet transform (8) can iteratively decompose an image (2D) into four components. Each iteration splits the image both horizontally and vertically into low-frequency (low pass) and high-frequency (high pass) components. Thus, four

components are generated: a high-pass/high-pass component consisting of mostly diagonal structure, a high-pass/low-pass component consisting mostly of vertical structures, a low-pass/high-pass component consisting mostly of horizontal structure, and a low-pass/low-pass component that represents a blurred version of the original image. Subsequent iterations then repeat the decomposition on the low-pass/low-pass component from the previous iteration. These subsequent iterations highlight broader diagonal, vertical, and horizontal textures. And for each component, we calculated the energy (referred to with a suffix P1) & entropy (referred to with a suffix P2) feature. A wavelet transform of a 3D signal can be achieved by applying the 1D wavelet transform along all the three directions (x,y,z). Features obtained at each level of decomposition are referred with suffix L (example: L1, L2) and level of decomposition is referred to with a prefix C (example: C1 to C9).

E. **Pixel Histogram Features:** the pixel intensity histogram h(a) is the number of pixels that occurred for brightness level “a” with brightness level on the x-axis. The probability distribution of the brightness P(a) can be calculated as well. Six features: mean, standard deviation, skewness, kurtosis, energy, and entropy were then incorporated.

Supplemental Table 4. Distribution of quantitative imaging features at various levels of concordance and redundancy limits (Rsq at ≥ 0.95) for regions identified by a) Radiologist marked and b) habitats converged (sphere) c) habitat converged (≤Median, ADC).

a) Radiologist marked

**Concordance & Dynamic Range with Redundancy Reduction (Rsq ≥ 0.95)** *Test-ReTest mpMRI: Number of features* **(Radiologist)**				
**CCC & DR & (Rsq ≥ 0.95)**	**ADC**	**ADCz**	**T2**	**T2z**
≥0.95	0	0	0	0
≥0.90	0	0	1	0
≥0.85	0	0	2	1
≥0.80	0	0	2	3
≥0.75	0	0	5	4
≥0.70	1	2	8	7
≥0.65	1	2	9	8

b) Habitat –Sphere converged

**Concordance & Dynamic Range with Redundancy Reduction (Rsq ≥ 0.95)** *Test-ReTest mpMRI: Number of features* **(Habitats) *** Dependent *****				
**CCC & DR & (Rsq ≥ 0.95)**	**ADC**	**ADCz**	**T2**	**T2z**
≥0.95	0	0	0	1
≥0.90	0	0	1	1
≥0.85	1	0	3	3
≥0.80	1	1	4	6
≥0.75	1	2	5	8
≥0.70	3	3	6	9
≥0.65	6	5	8	10

C)
Habitat – 50% (LTE Median ADC map): Rsq 0.95

**Concordance & Dynamic Range & Rsq ≥ 0.95 (** dependent **)** *Test-ReTest mpMRI: Number of features* **(Habitat 50%)**				
**CCC & DR &**	**ADC**	**ADCz**	**T2**	**T2z**
≥0.95	0	0	2	1
≥0.90	0	0	2	1
≥0.85	0	0	2	1
≥0.80	0	0	2	2
≥0.75	0	0	2	2
≥0.70	0	0	5	2
≥0.65	1	1	6	3

Supplemental Table 5. Radiomic Features that show concordance and non-redundancy in the test-retest cohort **(**CCC& DR >= 0.65; Rsq ≥ 0.95) for a) radiologist marked region, and b) habitat – Sphere (15mm) converged c) habitat (≤Median ADC) region.

a) Radiologist marked regions

**ADC Features (Radiologist): CCC& DR >= 0.65; Rsq ≥ 0.95**		
	**ADC (Radiologist):** 1 feature	**ADCz (Radiologist):** 2 features
	F9:Stat-90th-percentile	F96:avgCooc_3D_Difference-var<br>*Shape& Size:*<br>F88:Centre-of-mass-shift-(mm)

**T2 Features (Radiologist): CCC& DR >= 0.65;Rsq ≥ 0.95**		
	**T2 (Radiologist):** 9 features	**T2z (Radiologist):** 8 features
	F138:GLSZM_Large-zone-low-grey-level-emphasis<br>F149:NGTDM_Contrast<br>F10:Stat-Max-grey-level<br>F150:NGTDM_Busyness<br>F107:avgCooc_3D_Inv-diff-mom-norm<br>F284:3D-Wave-P1-L2-C4-<br>F115:avgCooc_3D_Second-measure-of-information-correlation<br>*Size& Shape:*<br>F47:Vol-at-Int-fraction-diff<br>F43:Vol-at-Int-Fraction-10	F19:Stat-Root-Mn-Sq<br>F149:NGTDM_Contrast<br>F10:Stat-Max-grey-level<br>F12:Stat-range<br>F107:avgCooc_3D_Inv-diff-mom-norm-<br>F171:3D-LawsF-L5-R5-R5-<br>*Size& Shape:*<br>F47:Vol-at-Int-fraction-diff-<br>F43:Vol-at-Int-Fraction-10-

b. Habitat regions

**T2 Features (Habitats) : CCC& DR >= 0.65;Rsq ≥ 0.95**		
	**T2 (Habitats): 8 features**	**T2z (Habitats): 10 features**
	F124:avg_3D_GLN-(Grey-level-non-uniformity)<br>F231:3D-LawsF-R5-L5-R5<br>F300:3D-Wave-P1-L2-C12C<br>F140:GLSZM_Grey-level-non-uniformity<br>F115:avgCooc_3D_Second-measure-of-information-correlation<br>F114:avgCooc_3D_First-measure-of-information-correlation<br>F148:NGTDM_Coarseness<br><br>*Shape & Size:*<br>F54:Surface-area-(mm^2)	F115:avgCooc_3D_Second-measure-of-information-correlation<br>F97:avgCooc_3D_Difference-entropy<br>F41:Min-hist-Gradient<br>F140:GLSZM_Grey-level-non-uniformity<br>F231:3D-LawsF-R5-L5-R5<br>F114:avgCooc_3D_First-measure-of-information-correlation<br>F87:Weighted-CoM_z-(mm)<br>F148:NGTDM_Coarseness<br>*Shape & Size:*<br>F54:Surface-area-(mm^2)<br>F88:Centre-of-mass-shift-(mm)

**ADC Features (Habitats): CCC& DR >= 0.65; Rsq ≥ 0.95**		
	**ADC (Habitats): 6 features**	**ADCz (Habitats): 5 features**
	F171:3D-LawsF-L5-R5-R5<br>F140:GLSZM_Grey-level-non-uniformity<br>F124:avg_3D_GLN-(Grey-level-non-uniformity)<br>F148:NGTDM_Coarseness<br>F246:3D-LawsF-R5-R5-R5<br>*Shape&Size:*<br>F54:Surface-area-(mm^2)	F140:GLSZM_Grey-level-non-uniformity-<br>F7:Stat-Min-grey-level<br>F115:avgCooc_3D_Second-measure-of-information-correlation<br>F148:NGTDM_Coarseness-<br>*Shape & Size:*<br>F54:Surface-area-(mm^2)-

c)

**T2 Features (Habitat 50%): CCC& DR >= 0.65;Rsq ≥ 0.95**		
	**T2 (Hab-50):** 6 features	**T2z (Hab-50):** 3 features
	F113:avgCooc_3D_Cluster-prominence-ADC-T2<br>F282:3D-Wave-P1-L2-C3-ADC-T2<br>F290:3D-Wave-P1-L2-C7-ADC-T2<br>F149:NGTDM_Contrast-ADC-T2<br>F18:Stat-ENERGY-ADC-T2<br>F40:Max-hist-Gradient-grey-level-ADC-T2	F152:NGTDM_Strength-T2z<br>F19:Stat-Root-Mn-Sq-T2z<br>F149:NGTDM_Contrast-T2z

**ADC Features (Habitat 50%): CCC& DR >= 0.65;Rsq ≥ 0.95**		
	**ADC (Hab50): 1** features	**ADCz (Hab50):** 1 features
	F10:Stat-Max-grey-level-ADC	F10:Stat-Max-grey-level-ADC

REFERENCES:

1. D.-H Xu ASK, J.D. Furst, and D.S. Raicu. Run-length encoding for volumetric texture. the IASTED Int'l Conf on Visualization, Imaging and Image Processing2004.
2. Tang X. Texture information in run-length matrices. IEEE Transactions on Image Processing. 1998;7(11):1602-9.
3. A.S. Kurani D-HX, J.D. Furst, and D.S. Raicu. Co-occurrence matrices for volumetric data. 7th IASTED Int'l Conf on Computer Graphics and Imaging2004.
4. Mokji M.M ABSAR. Gray Level Co-Occurrence Matrix Computation Based On Haar Wavelet. IEEE Computer Graphics, Imaging and Visualisation (CGIV '07): IEEE Aug 2007; p. 273-9.
5. V.A. Kovalev FK, H.-J Gertz, and D.Y. von Cramon. Three-dimensional texture analysis of MRI brain datasets. IEEE Trans on Medical Imaging. 2001;20(5):424-33.
6. Laws K. Texture Image Segmentation. Los Angeles: University of South California 1980.
7. Benke K K CDaSDR. A study of the effect of image quality on texture energy measures. Meas Sci Technol. 1994;5:400-7.
8. K. Jafari-Khouzani HS-Z, K. Elisevich, and S. Patel. Comparison of 2D and 3D wavelet features for the lateralization. In Proc of SPIE Medical Imaging 2004: Physiology, Function and Structure from Medical Images. 2004;5369:593-601.
